# Supplementary material for: Metformin Prevents Key Mechanisms of Obesity-Related Complications in Visceral White Adipose Tissue of Obese Pregnant Mice
Source: Nutrients. 2022 May 30;14(11):2288. doi: 10.3390/nu14112288 (PMC9182976; doi:10.3390/nu14112288)
Supplement: Supplementary file 1 [file nutrients-14-02288-s001.zip › nutrients-1702985-supplementary.pdf]

Supplementary Material

Metformin prevents key mechanisms of obesity-related complications in visceral white adipose tissue of obese pregnant mice

Katrin Schmitz, Eva-Maria Turnwald, Tobias Kretschmer, Ruth Janoschek, Inga Bae-Gartz, Kathrin

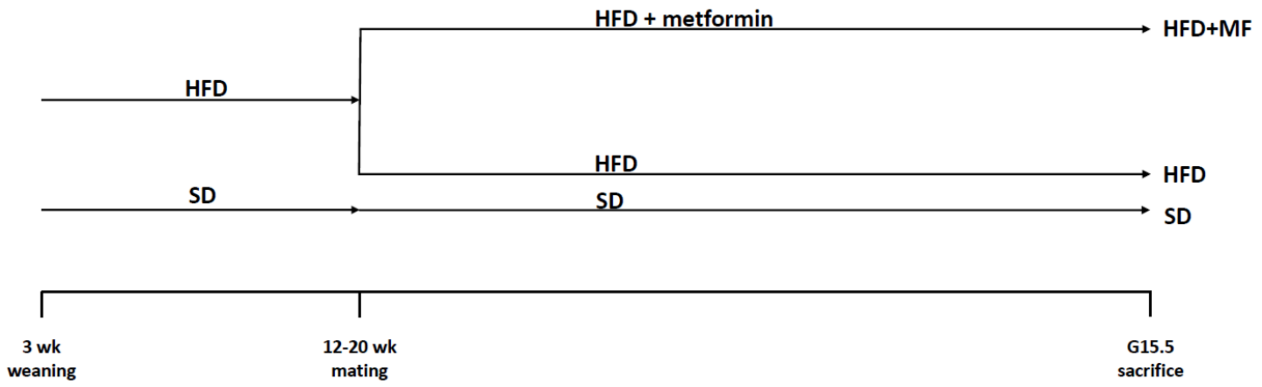

Figure S1. Schematic overview of the mouse groups used in this study. Female mice received either a SD or HFD after weaning until mating. After mating, the control group continued to receive the SD until the end of the experiment. The HFD group was randomly split in two subgroups after mating, while one subgroup continued to feed on HFD until sacrifice at G15.5 (HFD), and one subgroup received HFD plus metformin until G15.5 (HFD + MF). SD = standard diet; HFD = high fat diet; MF = metformin; G = gestational day; wk = week.

Table S1. Details on mouse diets used in this study. ME = metabolizable energy; SD = standard diet; HFD = high fat diet.

	SD	HFD
Company	Ssniff (Soest, Germany)	Altromin (Lage, Germany)
Catalog number	V1534 – R/M-Maintenance	C1057, modified
Metabolizable energy [kcal/kg]	3225	5237
Fat [% of ME]	9	60
Protein [% of ME]	33	16
Carbohydrates [% of ME]	58	24

Voßbrecher, Merlin D Kammerer, Angela Köninger, Alexandra Gellhaus, Marion Handwerk, Maria Wohlfarth, Dirk Gründemann, Eva Hucklenbruch-Rother, Jörg Dötsch, Sarah Appel

Summary tables from proteomic screen of pgWAT at the middle of last trimester

Proteins named in the discussion are highlighted in each table.

Table S2: Significantly and relevantly upregulated proteins in pgWAT at G15.5 in HFD vs SD. 53 proteins are significantly (qvalue < 0.05) and relevantly (fold change > 1.5) upregulated in obese (HFD) compared to lean dams (SD). Protein ID, protein name, gene, q-value (p-value corrected for multiple testing), fold change and valid values of both groups (= number of samples in which a protein was detected) are shown. The table is sorted according to fold change. 5 samples were analyzed per group.

Protein ID	Protein name	Gene	q-Value	Fold change	Valid values HFD	Valid values SD
**P27601**	Guanine nucleotide-binding protein subunit alpha-13	Gna13	< 0,01	6,92	5	0
**Q6PGH2**	Hematological and neurological expressed 1-like protein	Hn1l	< 0,05	5,72	5	0
**Q0PD20**	Ras-related protein Rab-34	Rab34	< 0,01	5,04	5	0
**A0A0R4J0F6**	Cyclin-G-associated kinase	Gak	< 0,0005	4,27	5	0
**Q9WTQ5**	A-kinase anchor protein 12	Akap12	< 0,01	4,23	5	5
**Q9Z0F7**	Gamma-synuclein	Sncg	< 0,05	4,03	5	5
**P26645**	Myristoylated alanine-rich C-kinase substrate	Marcks	< 0,05	4,03	5	5
**Q3THG9**	Alanyl-tRNA editing protein Aarsd1	Aarsd1	< 0,0005	3,88	5	0
**Q8CG16**	Complement C1r-A subcomponent	C1ra	< 0,05	3,83	5	0
**Q6PE70**	Integrin beta	Itgb5	< 0,001	3,35	5	0
**Q8K0C9**	GDP-mannose 4,6 dehydratase	Gmds	< 0,05	3,27	5	0
**E9PUM4**	Talin-2	Tln2	< 0,01	3,20	5	5
**E9QA16**	Caldesmon 1	Cald1	< 0,05	2,98	5	5
**Q8BH58**	TIP41-like protein	Tiprl	< 0,05	2,90	5	0
**P06728**	**Apolipoprotein A-IV**	**Apoa4**	**< 0,0005**	**2,73**	**5**	**5**
**Q3UH59**	Myosin-10	Myh10	< 0,05	2,69	5	5
**O88492**	Perilipin-4	Plin4	< 0,05	2,61	5	5
**P17439**	Glucosylceramidase	Gba	< 0,01	2,49	5	0
**Q9DCC5**	Chromobox protein homolog 3	Cbx3	< 0,05	2,32	5	5
**Q63918**	Serum deprivation-response protein	Sdpr	< 0,05	2,31	5	5
**P10107**	Annexin A1	Anxa1	< 0,001	2,16	5	5
**P63024**	Vesicle-associated membrane protein 3	Vamp3	< 0,05	2,15	5	5
**B1AQF4**	Dual specificity protein phosphatase 3	Dusp3	< 0,05	2,13	5	5
**Q9Z0P4**	Paralemmin-1	Palm	< 0,05	2,10	5	5
**Q91VJ2**	Protein kinase C delta-binding protein	Prkcdbp	< 0,05	2,09	5	5
**Q9DBX6**	**Cytochrome P450 2S1**	**Cyp2s1**	**< 0,05**	**2,09**	**5**	**5**
**Q00898**	Alpha-1-antitrypsin 1-5	Serpina1e	< 0,05	2,05	5	5
**P07356**	Annexin A2	Anxa2	< 0,01	2,04	5	5
**Q61792**	LIM and SH3 domain protein 1	Lasp1	< 0,05	2,04	5	5
**Q8BGD9**	Eukaryotic translation initiation factor 4B	Eif4b	< 0,05	2,01	5	5
**Q8BJW6**	Eukaryotic translation initiation factor 2A	Eif2a	< 0,05	1,98	5	5
**P27546**	Microtubule-associated protein 4	Map4	< 0,05	1,92	5	5
**O54724**	Polymerase I and transcript release factor	Ptrf	< 0,05	1,90	5	5
**A0A0R4J034**	Pyridoxal-dependent decarboxylase domaincontaining protein 1	Pdxdc1	< 0,05	1,89	5	5
**P31324**	cAMP-dependent protein kinase type II-beta regulatory subunit	Prkar2b	< 0,05	1,83	5	5
**Q61739**	Integrin alpha-6	Itga6	< 0,05	1,81	5	5
**P48036**	Annexin A5	Anxa5	< 0,05	1,79	5	5

**Q78IS1**	Transmembrane emp24 domain-containing protein 3	Tmed3	< 0,05	1,75	5	5
**E9Q8I9**	Protein furry homolog	Fry	< 0,05	1,74	5	0
**J3QNU6**	Beta-arrestin-1	Arrb1	< 0,05	1,73	5	5
**Q8R2Y2**	Cell surface glycoprotein MUC18	Mcam	< 0,05	1,70	5	5
**F8VQJ3**	Laminin subunit gamma-1	Lamc1	< 0,05	1,70	5	5
**Q6IRU2**	Tropomyosin alpha-4 chain	Tpm4	< 0,05	1,66	5	5
**Q9JIW9**	Ras-related protein Ral-B	Ralb	< 0,05	1,64	5	5
**A2AVJ7**	Ribosome-binding protein 1	Rrbp1	< 0,05	1,63	5	5
**P20152**	Vimentin	Vim	< 0,05	1,60	5	5
**F7AAP4**	Calcium-transporting ATPase	Atp2b4	< 0,05	1,59	5	5
**A3KGU7**	Spectrin alpha chain, non-erythrocytic 1	Sptan1	< 0,05	1,58	5	5
**E9Q616**	AHNAK nucleoprotein (desmoyokin)	Ahnak	< 0,05	1,54	5	5
**Q62261**	Spectrin beta chain, non-erythrocytic 1	Sptbn1	< 0,05	1,54	5	5
**P47955**	60S acidic ribosomal protein P1	Rplp1	< 0,05	1,54	5	5
**Q9CWF2**	Tubulin beta-2B chain	Tubb2b	< 0,05	1,53	5	5
**F8WIT2**	Annexin	Anxa6	< 0,05	1,53	5	5

Table S3: Significantly and relevantly downregulated proteins in pgWAT at G15.5 in HFD vs SD. 143 proteins are significantly (q-value < 0.05) and relevantly (fold change > 1.5) downregulated in obese (HFD) compared to lean dams (SD). Protein ID, protein name, gene, q-value (p-value corrected for multiple testing), fold change and valid values of both groups (= number of samples in which a protein was detected) are shown. The table is sorted according to fold change. 5 samples were analyzed per group.

Protein ID	Protein name	Gene	q-Value	Fold change	Valid values HFD	Valid values SD
**Q62264**	Thyroid hormone-inducible hepatic protein	Thrsp	< 0,01	-12,47	5	5
**Q9JK42**	**[Pyruvate dehydrogenase (acetyl-transferring)] kinase isozyme 2, mitochondrial**	**Pdk2**	**< 0,0005**	**-8,76**	**0**	**5**
**Q3V117**	ATP-citrate synthase	Acly	< 0,0005	-8,52	5	5
**F8VPN4**	4-alpha-glucanotransferase	Agl	< 0,05	-8,32	5	5
**Q5SWU9**	Acetyl-CoA carboxylase 1	Acaca	< 0,001	-7,32	5	5
**Q80Y98**	Phospholipase DDHD2	Ddhd2	< 0,01	-6,98	5	5
**Q99J39**	**Malonyl-CoA decarboxylase, mitochondrial**	**Mlycd**	**< 0,01**	**-6,34**	**0**	**5**
**Q8R2U6**	Diphosphoinositol polyphosphate phosphohydrolase 2	Nudt4	< 0,05	-5,36	0	5
**Q3TJZ6**	Protein FAM98A	Fam98a	< 0,05	-5,17	0	5
**A2AQN4**	Acetyl-coenzyme A synthetase, cytoplasmic	Acss2	< 0,01	-5,15	5	5
**A0A1B0GT63**	Transmembrane protein 143	Tmem143	< 0,01	-5,09	0	5
**Q9CQ20**	Mid1-interacting protein 1	Mid1ip1	< 0,05	-4,85	0	5
**Q8BGS7**	Choline/ethanolaminephosphotransferase 1	Cept1	< 0,05	-4,81	0	5
**P19096**	Fatty acid synthase	Fasn	< 0,0005	-4,64	5	5
**Q64521**	Glycerol-3-phosphate dehydrogenase, mitochondrial	Gpd2	< 0,0005	-4,58	5	5
**Q9CXI0**	2-methoxy-6-polyprenyl-1,4-benzoquinol methylase, mitochondrial	Coq5	< 0,05	-4,29	0	5
**A2AQZ2**	Phytanoyl-CoA dioxygenase domain-containing protein 1	Phyhd1	< 0,05	-4,15	0	5
**Q9CR26**	Vacuolar protein sorting-associated protein VTA1 homolog	Vta1	< 0,05	-4,05	0	5
**B7ZMP1**	Probable Xaa-Pro aminopeptidase 3	Xpnpep3	< 0,05	-3,93	0	5
**G3UZY2**	Thioredoxin, mitochondrial	Txn2	< 0,01	-3,88	0	5
**Q8K0Z7**	Translational activator of cytochrome c oxidase 1	Taco1	< 0,0005	-3,83	0	5
**P06801**	NADP-dependent malic enzyme	Me1	< 0,01	-3,78	5	5

**D3Z4P2**	VIP36-like protein	Lman2l	< 0,01	-3,47	0	5
**Q8BHE8**	Uncharacterized protein C2orf47 homolog, mitochondrial	Maip1	< 0,01	-3,41	0	5
**P70444**	BH3-interacting domain death agonist	Bid	< 0,01	-3,25	0	5
**Q9D2R0**	Acetoacetyl-CoA synthetase	Aacs	< 0,05	-3,17	5	5
**Q99LB7**	Sarcosine dehydrogenase, mitochondrial	Sardh	< 0,05	-3,17	5	5
**O08528**	Hexokinase-2	Hk2	< 0,05	-3,07	5	5
**P13516**	Acyl-CoA desaturase 1	Scd1	< 0,01	-3,07	5	5
**A2AKN9**	Major urinary protein 6	Mup4	< 0,01	-3,04	5	5
**P52196**	Thiosulfate sulfurtransferase	Tst	< 0,0005	-2,99	5	5
**Q9CZB0**	Succinate dehydrogenase cytochrome b560 subunit, mitochondrial	Sdhc	< 0,05	-2,97	5	5
**A0A1L1SRX2**	AMP deaminase 3	Ampd3	< 0,05	-2,93	5	5
**Q8JZU2**	Tricarboxylate transport protein, mitochondrial	Slc25a1	< 0,01	-2,92	5	5
**Q8VHY0**	Chondroitin sulfate proteoglycan 4	Cspg4	< 0,05	-2,90	5	5
**A0A0R4J0L6**	28S ribosomal protein S35, mitochondrial	Mrps35	< 0,05	-2,88	0	5
**A2A848**	Peroxisomal acyl-coenzyme A oxidase 1	Acox1	< 0,05	-2,87	5	5
**E9PXY1**	Cullin-4B	Cul4b	< 0,05	-2,80	0	5
**A0A140T8R8**	DCN1-like protein	Dcun1d1	< 0,05	-2,76	0	5
**Q8R086**	Sulfite oxidase, mitochondrial	Suox	< 0,0005	-2,72	5	5
**Q9D024**	Coiled-coil domain-containing protein 47	Ccdc47	< 0,05	-2,71	5	5
**Q9D6M3**	Mitochondrial glutamate carrier 1	Slc25a22	< 0,001	-2,69	5	5
**Q922B1**	O-acetyl-ADP-ribose deacetylase MACROD1	Macrod1	< 0,05	-2,68	5	5
**P30115**	Glutathione S-transferase A3	Gsta3	< 0,05	-2,67	5	5
**P40142**	Transketolase	Tkt	< 0,05	-2,66	5	5
**Q9DCW5**	Cytochrome c oxidase subunit 6A, mitochondrial	Cox6a1	< 0,05	-2,65	5	5
**S4R225**	WD repeat-containing protein 13	Wdr13	< 0,05	-2,64	0	5
**Q8K3J1**	NADH dehydrogenase [ubiquinone] iron-sulfur protein 8, mitochondrial	Ndufs8	< 0,05	-2,64	5	5
**Q8K411**	Presequence protease, mitochondrial	Pitrm1	< 0,05	-2,63	5	5
**Q9D6Y9**	1,4-alpha-glucan-branching enzyme	Gbe1	< 0,0005	-2,60	5	5
**Q9D023**	Mitochondrial pyruvate carrier 2	Mpc2	< 0,05	-2,60	5	5
**Q8CFR5**	Dystrobrevin	Dtna	< 0,05	-2,59	0	5
**Q99LY9**	NADH dehydrogenase [ubiquinone] iron-sulfur protein 5	Ndufs5	< 0,05	-2,59	5	5
**A0A0R3P9C8**	NADH dehydrogenase [ubiquinone] 1 alpha subcomplex subunit 9, mitochondrial	Ndufa9	< 0,05	-2,59	5	5
**Q9CYV5**	Transmembrane protein 135	Tmem135	< 0,01	-2,58	0	5
**A0A0B4J1G1**	Low affinity immunoglobulin gamma Fc region receptor II	Fcgr2b	< 0,05	-2,56	0	5
**Q3URE1**	Acyl-CoA synthetase family member 3, mitochondrial	Acsf3	< 0,05	-2,56	5	5
**J3QMN4**	Thioredoxin reductase 2, mitochondrial	Txnrd2	< 0,01	-2,55	5	5
**A0A0R4J023**	Methylglutaconyl-CoA hydratase, mitochondrial	Auh	< 0,05	-2,52	5	5
**Q9QZA0**	Carbonic anhydrase 5B, mitochondrial	Ca5b	< 0,01	-2,48	5	5
**Q8CAY6**	Acetyl-CoA acetyltransferase, cytosolic	Acat2	< 0,05	-2,47	5	5
**Q8R164**	Valacyclovir hydrolase	Bphl	< 0,05	-2,46	5	5
**Q9D0I4**	Syntaxin-17	Stx17	< 0,05	-2,44	0	5
**O70589**	Peripheral plasma membrane protein CASK	Cask	< 0,05	-2,43	0	5
**Q9CPR5**	39S ribosomal protein L15, mitochondrial	Mrpl15	< 0,05	-2,42	0	5
**Q9CQZ5**	NADH dehydrogenase [ubiquinone] 1 alpha subcomplex subunit 6	Ndufa6	< 0,01	-2,39	5	5

**E9QLB2**	Lysophospholipase-like protein 1	Lyplal1	< 0,05	-2,34	5	5
**Q9DBL1**	Short/branched chain specific acyl-CoA dehydrogenase, mitochondrial	Acadsb	< 0,01	-2,33	5	5
**Q9CPQ1**	Cytochrome c oxidase subunit 6C	Cox6c	< 0,05	-2,30	5	5
**E9QNL5**	Sulfotransferase	Sult1a1	< 0,05	-2,29	5	5
**Q91VM9**	Inorganic pyrophosphatase 2, mitochondrial	Ppa2	< 0,05	-2,28	5	5
**Q9CR62**	Mitochondrial 2-oxoglutarate/malate carrier protein	Slc25a11	< 0,0005	-2,27	5	5
**Q8BWM0**	Prostaglandin E synthase 2	Ptges2	< 0,01	-2,27	5	5
**G5E8R3**	Pyruvate carboxylase	Pcx	< 0,05	-2,25	5	5
**F8WIU1**	UPF0687 protein C20orf27 homolog	1700037 H04Rik	< 0,05	-2,25	5	5
**Q9CQC7**	NADH dehydrogenase [ubiquinone] 1 beta subcomplex subunit 4	Ndufb4	< 0,01	-2,25	5	5
**Q8K0C4**	Lanosterol 14-alpha demethylase	Cyp51a1	< 0,05	-2,23	0	5
**Q505D7**	**Optic atrophy 3 protein homolog**	**Opa3**	**< 0,05**	**-2,20**	**0**	**5**
**Q9ET01**	Glycogen phosphorylase, liver form	Pygl	< 0,01	-2,17	5	5
**A0A0R4J1R7**	Pterin-4-alpha-carbinolamine dehydratase 2	Pcbd2	< 0,05	-2,17	5	5
**P0DN34**	NADH dehydrogenase [ubiquinone] 1 beta subcomplex subunit 1	Ndufb1	< 0,01	-2,14	5	5
**D3YUM1**	NADH dehydrogenase [ubiquinone] flavoprotein 1, mitochondrial	Ndufv1	< 0,05	-2,10	5	5
**O88696**	ATP-dependent Clp protease proteolytic subunit, mitochondrial	Clpp	< 0,01	-2,10	5	5
**Q9ERS2**	NADH dehydrogenase [ubiquinone] 1 alpha subcomplex subunit 13	Ndufa13	< 0,05	-2,10	5	5
**Q99JR6**	Nicotinamide/nicotinic acid mononucleotide adenylyltransferase 3	Nmnat3	< 0,05	-2,09	0	5
**Q9D7J9**	Enoyl-CoA hydratase domain-containing protein 3, mitochondrial	Echdc3	< 0,01	-2,08	5	5
**Q99LS3**	Phosphoserine phosphatase	Psph	< 0,05	-2,07	5	5
**Q91WD5**	NADH dehydrogenase [ubiquinone] iron-sulfur protein 2, mitochondrial	Ndufs2	< 0,05	-2,07	5	5
**Q9CQQ7**	ATP synthase F(0) complex subunit B1, mitochondrial	Atp5f1	< 0,01	-2,06	5	5
**A0A1B0GT92**	Glycogen [starch] synthase, muscle	Gys1	< 0,05	-2,03	5	5
**Q99LC3**	NADH dehydrogenase [ubiquinone] 1 alpha subcomplex subunit 10, mitochondrial	Ndufa10	< 0,0005	-2,02	5	5
**Q14DH7**	Acyl-CoA synthetase short-chain family member 3, mitochondrial	Acss3	< 0,05	-2,01	5	5
**P56135**	ATP synthase subunit f, mitochondrial	Atp5j2	< 0,05	-2,00	5	5
**Q9CQJ8**	NADH dehydrogenase [ubiquinone] 1 beta subcomplex subunit 9	Ndufb9	< 0,05	-2,00	5	5
**Q7TMG8**	Protein NipSnap homolog 2	Gbas	< 0,01	-1,99	5	5
**P00405**	Cytochrome c oxidase subunit 2	mt-Co2	< 0,05	-1,99	5	5
**Q99P31**	Hsp70-binding protein 1	Hspbp1	< 0,05	-1,93	0	5
**Q91ZJ5**	UTP--glucose-1-phosphate uridylyltransferase	Ugp2	< 0,05	-1,91	5	5
**Q8CHT0**	Delta-1-pyrroline-5-carboxylate dehydrogenase, mitochondrial	Aldh4a1	< 0,05	-1,91	5	5
**A0A0A0MQ68**	Glutaryl-CoA dehydrogenase, mitochondrial	Gcdh	< 0,05	-1,91	5	5
**Q9DBL7**	Bifunctional coenzyme A synthase	Coasy	< 0,05	-1,90	5	5
**P35486**	Pyruvate dehydrogenase E1 component subunit alpha, somatic form, mitochondrial	Pdha1	< 0,05	-1,88	5	5
**Q9DCT2**	NADH dehydrogenase [ubiquinone] iron-sulfur protein 3, mitochondrial	Ndufs3	< 0,05	-1,87	5	5

**P03911**	NADH-ubiquinone oxidoreductase chain 4	mt-Nd4	< 0,05	-1,87	5	5
**P52480**	Pyruvate kinase PKM	Pkm	< 0,05	-1,86	5	5
**Q9QZD8**	Mitochondrial dicarboxylate carrier	Slc25a10	< 0,05	-1,85	5	5
**Q8BGC4**	Zinc-binding alcohol dehydrogenase domaincontaining protein 2	Zadh2	< 0,05	-1,82	5	5
**Q91VT4**	Carbonyl reductase family member 4	Cbr4	< 0,05	-1,82	5	5
**Q8CG76**	Aflatoxin B1 aldehyde reductase member 2	Akr7a2	< 0,05	-1,81	5	5
**P97372**	Proteasome activator complex subunit 2	Psme2	< 0,05	-1,80	5	5
**P03888**	NADH-ubiquinone oxidoreductase chain 1	mt-Nd1	< 0,05	-1,80	5	5
**Q9QYR9**	Acyl-coenzyme A thioesterase 2, mitochondrial	Acot2	< 0,05	-1,80	5	5
**Q9D0M3**	Cytochrome c1, heme protein, mitochondrial	Cyc1	< 0,01	-1,79	5	5
**Q9DC61**	Mitochondrial-processing peptidase subunit alpha	Pmpca	< 0,05	-1,79	5	5
**P14142**	Solute carrier family 2, facilitated glucose transporter member 4	Slc2a4	< 0,05	-1,79	5	5
**P05202**	Aspartate aminotransferase, mitochondrial	Got2	< 0,05	-1,78	5	5
**Q9DCJ5**	NADH dehydrogenase [ubiquinone] 1 alpha subcomplex subunit 8	Ndufa8	< 0,05	-1,77	5	5
**Q9DB77**	Cytochrome b-c1 complex subunit 2, mitochondrial	Uqcrc2	< 0,01	-1,77	5	5
**Q925I1**	ATPase family AAA domain-containing protein 3	Atad3	< 0,05	-1,76	5	5
**Q8CGK3**	Lon protease homolog, mitochondrial	Lonp1	< 0,05	-1,74	5	5
**Q8K2B3**	Succinate dehydrogenase [ubiquinone] flavoprotein subunit, mitochondrial	Sdha	< 0,01	-1,72	5	5
**P06745**	Glucose-6-phosphate isomerase	Gpi	< 0,01	-1,72	5	5
**Q3UW66**	Sulfurtransferase	Mpst	< 0,05	-1,72	5	5
**P55302**	Alpha-2-macroglobulin receptor-associated protein	Lrpap1	< 0,05	-1,70	5	5
**P12382**	ATP-dependent 6-phosphofructokinase, liver type	Pfkl	< 0,01	-1,70	5	5
**Q6PB66**	Leucine-rich PPR motif-containing protein, mitochondrial	Lrpprc	< 0,05	-1,67	5	5
**Q9R1J0**	Sterol-4-alpha-carboxylate 3-dehydrogenase, decarboxylating	Nsdhl	< 0,05	-1,67	5	5
**Q9D172**	ES1 protein homolog, mitochondrial	D10Jhu81e	< 0,05	-1,64	5	5
**Q9DAR7**	m7GpppX diphosphatase	Dcps	< 0,01	-1,63	5	5
**P47738**	Aldehyde dehydrogenase, mitochondrial	Aldh2	< 0,05	-1,62	5	5
**Q8BQ47**	Protein canopy homolog 4	Cnpy4	< 0,001	-1,62	5	5
**Q9JMH6**	Thioredoxin reductase 1, cytoplasmic	Txnrd1	< 0,01	-1,60	5	5
**Q9D1D4**	Transmembrane emp24 domain-containing protein 10	Tmed10	< 0,05	-1,60	5	5
**Q9D6J6**	NADH dehydrogenase [ubiquinone] flavoprotein 2, mitochondrial	Ndufv2	< 0,05	-1,60	5	5
**O08749**	Dihydrolipoyl dehydrogenase, mitochondrial	Dld	< 0,05	-1,59	5	5
**P63325**	40S ribosomal protein S10	Rps10	< 0,05	-1,58	5	5
**Q8BGH2**	Sorting and assembly machinery component 50 homolog	Samm50	< 0,05	-1,58	5	5
**P15105**	Glutamine synthetase	Glul	< 0,05	-1,58	5	5
**Q03958**	Prefoldin subunit 6	Pfdn6	< 0,05	-1,56	5	5
**Q8R0Y6**	Cytosolic 10-formyltetrahydrofolate dehydrogenase	Aldh1l1	< 0,05	-1,55	5	5
**Q9CY27**	Very-long-chain enoyl-CoA reductase	Tecr	< 0,05	-1,54	5	5
**P28063**	Proteasome subunit beta type-8	Psmb8	< 0,05	-1,52	5	5
**Q9R0X4**	Acyl-coenzyme A thioesterase 9, mitochondrial	Acot9	< 0,05	-1,52	5	5

Table S4: Significantly and relevantly upregulated proteins in pgWAT at G15.5 in HFD+MF vs HFD. 58 proteins are significantly (q-value < 0.05) and relevantly (fold change > 1.5) upregulated in obese dams treated with metformin during pregnancy (HFD+MF) compared to obese dams (HFD). Protein ID, protein name, gene, q-value (p-value corrected for multiple testing), fold change and valid values of both groups (= number of samples in which a protein was detected) are shown. The table is sorted according to fold change. 4 HFD+MF samples and 5 HFD samples were analyzed.

Protein ID	Protein name	Gene	q-Value	Fold change	Valid values HFD+MF	Valid values HFD
**S4R211**	Serine-rich coiled-coil domain-containing protein 2	Ccser2	< 0,05	13,01	4	0
**Q9Z0Y1**	Dynactin subunit 3	Dctn3	< 0,01	8,53	4	0
**Q61555**	Fibrillin-2	Fbn2	< 0,05	8,00	4	0
**B1AZ15**	Cordon-bleu protein-like 1	Cobll1	< 0,05	7,94	4	0
**Q9CQR4**	Acyl-coenzyme A thioesterase 13	Acot13	< 0,05	7,46	4	0
**Q99J39**	**Malonyl-CoA decarboxylase, mitochondrial**	**Mlycd**	**< 0,05**	**7,19**	**4**	**0**
**Q8K019**	Bcl-2-associated transcription factor 1	Bclaf1	< 0,05	5,71	4	0
**Q8K0D5**	Elongation factor G, mitochondrial	Gfm1	< 0,05	4,87	4	0
**D3YTQ3**	Heterogeneous nuclear ribonucleoprotein D-like	Hnrnpdl	< 0,05	4,80	4	0
**Q99JC1**	Ig lambda-2 chain C region	Iglc2	< 0,05	4,73	4	0
**Q9QUT0**	Ammonium transporter Rh type A	Rhag	< 0,05	4,69	4	0
**Q59IW6**	ABI gene family, member 3 (NESH)-binding protein	Abi3bp	< 0,05	4,44	4	0
**Q9JK42**	**[Pyruvate dehydrogenase (acetyl-transferring)] kinase isozyme 2, mitochondrial**	**Pdk2**	**< 0,05**	**4,35**	**4**	**0**
**Q9D0R8**	Protein LSM12 homolog	Lsm12	< 0,05	4,35	4	0
**Q6GQT1**	Alpha-2-macroglobulin-P	A2m	< 0,05	4,29	4	5
**G3X9Q2**	Guanine nucleotide-binding protein subunit gamma	Gng7	< 0,05	4,29	4	0
**P52623**	Uridine-cytidine kinase 1	Uck1	< 0,05	3,87	4	0
**A0A0R4J1Z3**	Transmembrane protein 33	Tmem33	< 0,05	3,86	4	0
**Q9CPU2**	NADH dehydrogenase [ubiquinone] 1 beta subcomplex subunit 2, mitochondrial	Ndufb2	< 0,05	3,71	4	0
**Q61584**	Fragile X mental retardation syndrome-related protein 1	Fxr1	< 0,05	3,57	4	0
**Q569Z5**	Probable ATP-dependent RNA helicase DDX46	Ddx46	< 0,05	3,55	4	0
**B1AUB9**	Nuclear factor 1	Nfia	< 0,05	3,48	4	0
**A0A0G2JER9**	DnaJ homolog subfamily B member 6	Dnajb6	< 0,05	3,36	4	0
**Q8CC86**	Nicotinate phosphoribosyltransferase	Naprt	< 0,05	3,35	4	0
**Q9D020**	Cytosolic 5-nucleotidase 3A	Nt5c3a	< 0,05	3,34	4	0
**F6RJ39**	Apoptotic chromatin condensation inducer in the nucleus	Acin1	< 0,05	3,34	4	0
**H3BL37**	Treacle protein	Tcof1	< 0,05	3,30	4	0
**Q3UE37**	Ubiquitin-conjugating enzyme E2 Z	Ube2z	< 0,05	3,21	4	0
**A0A0R4J1W7**	Cell division cycle protein 23 homolog	Cdc23	< 0,05	3,08	4	0
**Q9JIZ9**	Phospholipid scramblase 3	Plscr3	< 0,05	3,08	4	0
**Q3U422**	NADH dehydrogenase [ubiquinone] flavoprotein 3, mitochondrial	Ndufv3	< 0,05	3,04	4	5
**Q9D289**	Trafficking protein particle complex subunit 6B	Trappc6b	< 0,05	2,88	4	5
**D3Z2Z1**	CAP-Gly domain-containing linker protein 1	Clip1	< 0,05	2,69	4	0
**Q8VI75**	Importin-4	Ipo4	< 0,05	2,69	4	5
**Q9CPU4**	Microsomal glutathione S-transferase 3	Mgst3	< 0,05	2,66	4	5

**Q8C570**	mRNA export factor	Rae1	< 0,05	2,40	4	0
**Q3UID0**	SWI/SNF complex subunit SMARCC2	Smarcc2	< 0,05	2,39	4	5
**Q91V35**	Receptor-type tyrosine-protein phosphatase	Ptpra	< 0,05	2,38	4	0
**D3Z7P3**	Glutaminase kidney isoform, mitochondrial	Gls	< 0,05	2,31	4	5
**A0A0R4J0X5**	**Alpha-1-antitrypsin 1-3**	**Serpina1c**	**< 0,05**	**2,25**	**4**	**5**
**P30355**	Arachidonate 5-lipoxygenase-activating protein	Alox5ap	< 0,05	2,20	4	0
**A2AEM2**	Chloride channel CLIC-like protein 1	Clcc1	< 0,05	2,19	4	0
**Q91VS7**	Microsomal glutathione S-transferase 1	Mgst1	< 0,05	2,11	4	5
**P22599**	**Alpha-1-antitrypsin 1-2**	**Serpina1b**	**< 0,05**	**2,05**	**4**	**5**
**P06728**	**Apolipoprotein A-IV**	**Apoa4**	**< 0,05**	**1,91**	**4**	**5**
**A0A0R4J170**	Transcription activator BRG1	Smarca4	< 0,05	1,86	4	0
**Q3UIA2**	Rho GTPase-activating protein 17	Arhgap17	< 0,05	1,85	4	5
**Q99LC5**	Electron transfer flavoprotein subunit alpha, mitochondrial	Etfa	< 0,05	1,79	4	5
**Q9Z0U1**	Tight junction protein ZO-2	Tjp2	< 0,05	1,78	4	0
**Q505D7**	**Optic atrophy 3 protein homolog**	**Opa3**	**< 0,05**	**1,71**	**4**	**0**
**Q9Z204**	Heterogeneous nuclear ribonucleoproteins C1/C2	Hnrnpc	< 0,05	1,69	4	5
**Q91XD7**	Cysteine-rich with EGF-like domain protein 1	Creld1	< 0,05	1,64	4	5
**Q9DB20**	ATP synthase subunit O, mitochondrial	Atp5o	< 0,05	1,61	4	5
**Q9QYJ0**	DnaJ homolog subfamily A member 2	Dnaja2	< 0,05	1,61	4	5
**P16858**	**Glyceraldehyde-3-phosphate dehydrogenase**	**Gapdh**	**< 0,05**	**1,58**	**4**	**5**
**P21619**	Lamin-B2	Lmnb2	< 0,05	1,56	4	5
**P54822**	Adenylosuccinate lyase	Adsl	< 0,05	1,54	4	5
**Q62465**	Synaptic vesicle membrane protein VAT-1 homolog	Vat1	< 0,01	1,53	4	5

Table S5: Significantly and relevantly downregulated proteins in pgWAT at G15.5 in HFD+MF vs HFD. 45 proteins are significantly (q-value < 0.05) and relevantly (fold change > 1.5) downregulated in obese dams treated with metformin during pregnancy (HFD+MF) compared to obese dams (HFD). Protein ID, protein name, gene, q-value (p-value corrected for multiple testing), fold change and valid values of both groups (= number of samples in which a protein was detected) are shown. The table is sorted according to fold change. 4 HFD+MF samples and 5 HFD samples were analyzed.

Protein ID	Protein name	Gene	q-Value	Fold change	Valid values HFD+MF	Valid values HFD
**P20065**	Thymosin beta-4	Tmsb4x	< 0,05	-33,30	0	5
**A0A0U1RNP7**	**Perilipin-1**	**Plin1**	**< 0,01**	**-15,75**	**0**	**5**
**Q9ERT9**	Protein phosphatase 1 regulatory subunit 1A	Ppp1r1a	< 0,05	-9,09	0	5
**P58044**	Isopentenyl-diphosphate Delta-isomerase 1	Idi1	< 0,05	-5,57	0	5
**G5E870**	E3 ubiquitin-protein ligase TRIP12	Trip12	< 0,05	-3,60	0	5
**Q9Z0J0**	Epididymal secretory protein E1	Npc2	< 0,05	-3,49	4	5
**Q91XL1**	**Leucine-rich HEV glycoprotein**	**Lrg1**	**< 0,05**	**-3,40**	**4**	**5**
**Q62356**	Follistatin-related protein 1	Fstl1	< 0,05	-3,16	4	5
**Q9QXC1**	Fetuin-B	Fetub	< 0,05	-2,60	4	5
**Q8QZR5**	Alanine aminotransferase 1	Gpt	< 0,05	-2,39	4	5
**Q9DBX6**	**Cytochrome P450 2S1**	**Cyp2s1**	**< 0,05**	**-2,33**	**4**	**5**
**P51855**	Glutathione synthetase	Gss	< 0,05	-2,28	4	5
**P67984**	60S ribosomal protein L22	Rpl22	< 0,05	-2,07	4	5
**Q9D1X0**	Nucleolar protein 3	Nol3	< 0,05	-2,05	4	5
**Q9QZ06**	Toll-interacting protein	Tollip	< 0,05	-2,03	4	5
**Q9CXR1**	Dehydrogenase/reductase SDR family member 7	Dhrs7	< 0,05	-1,97	4	5

**P62897**	Cytochrome c, somatic	Cycs	< 0,05	-1,96	4	5
**Q9R099**	Transducin beta-like protein 2	Tbl2	< 0,05	-1,93	4	5
**Q9CQS8**	Protein transport protein Sec61 subunit beta	Sec61b	< 0,05	-1,90	4	5
**Q9ERE7**	LDLR chaperone MESD	Mesdc2	< 0,05	-1,89	4	5
**Q8R317**	Ubiquilin-1	Ubqln1	< 0,05	-1,77	4	5
**P63330**	Serine/threonine-protein phosphatase 2A catalytic subunit alpha isoform	Ppp2ca	< 0,05	-1,74	4	5
**Q9CQ65**	S-methyl-5-thioadenosine phosphorylase	Mtap	< 0,05	-1,72	4	5
**Q922Q8**	Leucine-rich repeat-containing protein 59	Lrrc59	< 0,05	-1,72	4	5
**P17563**	**Selenium-binding protein 1**	**Selenbp1**	**< 0,05**	**-1,71**	**4**	**5**
**P70296**	Phosphatidylethanolamine-binding protein 1	Pebp1	< 0,05	-1,71	4	5
**A0A0B4J1E7**	Importin subunit alpha-3	Kpna4	< 0,05	-1,70	4	5
**Q9Z1Z2**	Serine-threonine kinase receptor-associated protein	Strap	< 0,05	-1,69	4	5
**Q61035**	Histidine--tRNA ligase, cytoplasmic	Hars	< 0,05	-1,68	4	5
**O08915**	AH receptor-interacting protein	Aip	< 0,05	-1,67	4	5
**Q3UGR5**	Haloacid dehalogenase-like hydrolase domain-containing protein 2	Hdhd2	< 0,05	-1,67	4	5
**P68037**	Ubiquitin-conjugating enzyme E2 L3	Ube2l3	< 0,05	-1,66	4	5
**Q91YJ2**	Sorting nexin-4	Snx4	< 0,05	-1,64	4	5
**O35945**	Aldehyde dehydrogenase, cytosolic 1	Aldh1a7	< 0,05	-1,63	4	5
**P57759**	Endoplasmic reticulum resident protein 29	Erp29	< 0,05	-1,61	4	5
**E9Q137**	Testis-expressed gene 264	Tex264	< 0,05	-1,61	4	5
**Q9QZ88**	Vacuolar protein sorting-associated protein 29	Vps29	< 0,05	-1,58	4	5
**O70274**	Protein tyrosine phosphatase type IVA 2	Ptp4a2	< 0,05	-1,58	4	5
**Q9DAK9**	14 kDa phosphohistidine phosphatase	Phpt1	< 0,05	-1,57	4	5
**Q91V41**	Ras-related protein Rab-14	Rab14	< 0,05	-1,56	4	5
**O35598**	Disintegrin and metalloproteinase domain-containing protein 10	Adam10	< 0,05	-1,55	4	5
**Q07797**	Galectin-3-binding protein	Lgals3bp	< 0,05	-1,55	4	5
**Q8BKE6**	Cytochrome P450 20A1	Cyp20a1	< 0,05	-1,55	4	5
**Q8JZN1**	Macrophage galactose N-acetyl-galactosamine-specific lectin 2	Mgl2	< 0,05	-1,54	4	5
**Q02819**	Nucleobindin-1	Nucb1	< 0,05	-1,52	4	5
